# Decreased levels of circulating trimethylamine N-oxide alleviate cognitive and pathological deterioration in transgenic mice: a potential therapeutic approach for Alzheimer’s disease

**DOI:** 10.18632/aging.102352

**Published:** 2019-10-14

**Authors:** Qiang Gao, Yuan Wang, Xin Wang, Shuang Fu, Xin Zhang, Rui-Tao Wang, Xin Zhang

**Affiliations:** 1 Department of Geriatrics, The Second Affiliated Hospital of Harbin Medical University, Harbin 150086, China; 2 Department of Internal Medicine, Harbin Medical University Cancer Hospital, Harbin Medical University, Harbin 150081, China; 3 Department of Neurology, The First Affiliated Hospital of Harbin Medical University, Harbin 150001, China

**Keywords:** Alzheimer’s disease, APP/PS1 mice, trimethylamine-N-oxide, cognitive behavior, amyloid-β

## Abstract

Trimethylamine-N-oxide (TMAO), a metabolite of gut microbiota, has been implicated in the pathogenesis of Alzheimer’s disease (AD). However, the mechanisms by which TMAO influence cognitive and pathological processes in the AD have not been investigated. In this study, we found that the circulating TMAO levels displayed an age-related increase in both WT and APP/PS1 mice and association with AD-like behavioral and pathological profile. Reduced TMAO by 3,3-Dimethyl-1-butanol (DMB) treatment ameliorated the cognitive deterioration and long-term potentiation (LTP) in APP/PS1 mice. Moreover, DMB treatment also induced a decrease in the Amyloid-β (Aβ)_1-42_, β-secretase, and β-secretase-cleaved C-terminal fragment (βCTF) levels in the hippocampus. Finally, the effects obtained after treatment with DMB were accompanied by a reduction in circulating clusterin levels and hippocampal neuroinflammatory status in APP/PS1 mice. These findings demonstrate that elevated circulating TMAO during the aging process might deteriorate cognitive function and pathology in APP/PS1 mice.

## INTRODUCTION

Alzheimer’s disease (AD) is a progressive neurodegenerative disease that is the leading form of senile dementia characterized by the deposition of amyloid-β (Aβ) in the brain [1]. The misfolding, oligomerization and aggregation of the short Aβ peptides are essential events in the pathogenesis of AD, which will eventually lead to the subsequent formation of the extracellular plaques [2]. The Aβ peptides are derived from amyloid precursor protein (APP) by the actions of β- and γ-secretases. Aβ is omnipresent in vivo. But clinical studies have demonstrated that Aβ deposition is detected only in the cerebrum. The factors that accelerate Aβ accumulation and/or restrain its clearance in the cerebrum are still unknown [3]. However, several studies showed that alterations of the vascular system and vascular disorders, including stroke [4], atherosclerosis [5], and hypertension [6] are also involved in AD. Additionally, these alternations are considered as a risk factor for AD and are highly related to elevated brain amyloid deposition [6, 7].

Among peripheral cells, platelets, as an essential mediator of hemostasis and arterial thrombosis, could be a potential connection between vascular disorders and AD pathology [8]. High level of APP is expressed by platelets, the soluble fraction of which is stored in complete enzymatic machinery and release Aβ peptides in plasma on the basis of platelet activation [9, 10]. Platelets are therefore a key resource of Aβ in the bloodstream, which might contribute to the Aβ deposition in the cerebrum. Researchers activated the platelets in both in AD animal models and AD patients, which was proved to contribute to chronic neuroinflammation that consecutively leads to the accumulation of Aβ in the brain and vice versa [11]. Additionally, platelets of AD patients showed a high level of activation in resting states and high clusterin expression upon stimulation [12]. Pre-activated platelets, such as strongly enhanced integrin activation and spreading kinetics on fibrinogen surfaces, have been observed in aged AD transgenic mice. The hyperactivation of platelets leads to increase and worsen neuroinflammation [13]. Hence, platelets play a critical role in the AD pathological processes.

Accumulating evidence reveals that gut microbial metabolite trimethylamine-N-oxide (TMAO) has been implicated in AD pathogenesis [14]. Studies showed gut microbes, through the generation of TMAO, directly contribute to platelet hyperreactivity by enhancing the stimulus-dependent release of calcium ion from intracellular stores [15]. Moreover, the TMAO levels in cerebrospinal fluid of patients with AD dementia are higher than that in cognitively-unaffected individuals [16]. Furthermore, an integrated computational study revealed that TMAO was significantly associated with numerous aspects in AD, including disease susceptibility, cognitive decline, and the disease onset of AD [17]. Although the relationship between TMAO and AD pathology is becoming clear, the mechanisms whereby the TMAO contribute to pathological processes in AD have not been fully investigated. In this study, we examined cognitive declines, long-term potentiation (LTP), and pathological deterioration in male AD transgenic mice of different ages. Then we investigated the relationships between plasma TMAO levels and global index of AD-like behavioral and pathological profile in AD transgenic mice. To test the effect of TMAO on cognitive impairments and expression of amyloid-β, we also investigated the effect of a TMA formation inhibitor 3,3-Dimethyl-1-butanol (DMB) on cognitive function, pathologic changes and related secretases in transgenic AD mice. In addition, the ability of DMB to attenuate the release of clusterin in plasma and inflammatory status in the hippocampus were also evaluated.

## RESULTS

### Age-related senescence and cognitive declines in WT and APP/PS1 mice

**Figure 1:**
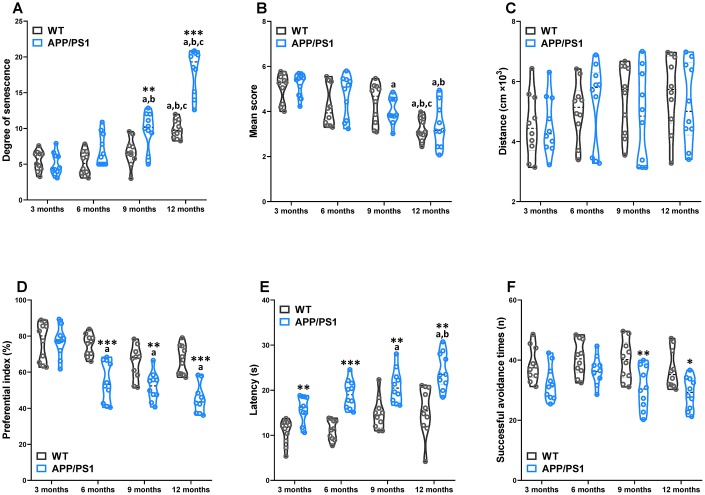
**Age-related senescence and cognitive impairments in WT and APP/PS1 mice.** The senescence degree (**A**), nest building score (**B**), and spontaneous locomotor activity (**C**) of WT and APP/PS1 mice. The preferential index (time on novel object C/(time on novel object C+time on sample object A)×100%) after training 1 hour (**D**) in the phase of novel object test. Latency, the first time that the mice crossed the former platform in the learning task of the Morris water maze test (**E**). The successful avoidance times in testing phase of shuttle box test (**F**). *P<0.05, **P<0.01, ***P<0.001, versus the age-matched WT mice, ^a^*P*<0.05, versus 3-month-old syngeneic mice, ^b^*P*<0.05, versus 6-month-old syngeneic mice, ^c^*P*<0.05, versus 9-month-old syngeneic mice by two-way repeated-measures analyses of variance with Tukey multiple comparisons tests. All values are means ± S.D. n=10.

The greatest known risk factor for Alzheimer’s disease is increasing age. For evaluation of the apparent senescence in APP/PS1 mice, a grading score system was employed (Figure 1A). Our results demonstrate that APP/PS1 mice exhibited a greater senescent degree than did WT mice at 9 and 12 months of age. In the APP/PS1 group, the significantly increased degree was observed since the 9-month-old. To evaluate spontaneous behavior of APP/PS1 mice, we used the locomotor activity test and nest building test respectively (Figure 1B and 1C). Our results demonstrate that the nest building score and locomotor activity of APP/PS1 mice did not differ significantly from the age-matched control littermates. The nest-building score within the group significantly decreased at 9 and 12 months of age in the APP/PS1 group, whereas it sharply decreased at 12 months of age in the WT group. To examine novel object recognition memory in these mice, we employed the novel object recognition test (Figure 1D). Except for the 3-month-old group, the preferential index was lower in the APP/PS1 group than in the WT group. To examine spatial memory in these mice, we used the Morris water maze test (Figure 1E). In the testing session, the APP/PS1 group exhibited a longer escape latency than did the WT group beginning on 3-month-old; however, there was no significant difference in the number of plate crossings, the time in the target quadrant, and swimming speed (data not shown) in APP/PS1 transgenic mice compared with WT mice. Finally, the shuttle-box test was used to evaluate active avoidance in mice in the present study (Figure 1F). Successful avoidance times in the APP/PS1 group were significantly decreased beyond WT group levels beginning on 9-month-old. These data indicate that learning and memory were age-related declines in APP/PS1 mice. The results of a Spearman correlation analysis showed that among the circulating TMAO levels, degree of senescence, nest building, spontaneous locomotor activity, object recognition memory, spatial learning and memory, and active avoidance in WT and APP/PS1 mice. TMAO levels were correlated with the degree of senescence, nest building, object recognition memory, spatial learning and memory, and active avoidance (Supplementary Figure 1).

### Age-related reduction of long-term potentiation in WT and APP/PS1 mice

**Figure 2:**
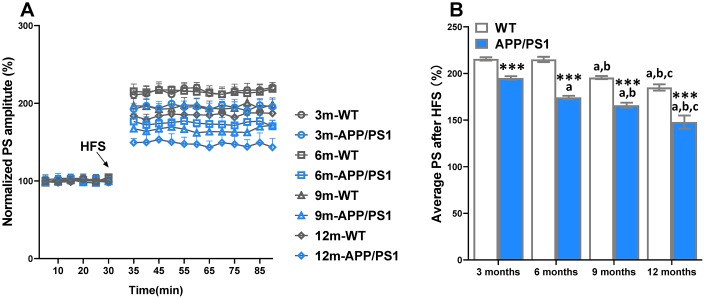
**Age-related decline of long-term potentiation (LTP) in APP/PS1 mice.** The magnitude of LTP in WT and APP/PS1 mice (**A**). High-frequency stimulation (HFS) was given at the 30 minutes of population spike (PS) baseline recording, and PS was recorded for the following 60 minutes. The average PS amplitudes in the control period were normalized as 100%, and the PS amplitudes at every point were normalized to them. Summary of average PS amplitude (31–90 minutes) in WT and APP/PS1 mice (**B**). ***P<0.001, versus the age-matched WT mice, ^a^*P*<0.05, versus 3-month-old syngeneic mice, ^b^*P*<0.05, versus 6-month-old syngeneic mice, ^c^*P*<0.05, versus 9-month-old syngeneic mice by one-way ANOVA analysis followed by Dunnett’s post hoc test. All values are means ± S.D. n=10.

LTP has been indorsed as a putative neuromechanism of associative memory formation and storage, and well associated with cognition ability [18]. The average PS amplitude after HFS within the group significantly decreased beginning on 3-month-old APP/PS1 group, whereas it sharply decreased at 9 and 12 months of age in the WT group (Figure 2). Compared with WT, the PS amplitude of APP/PS1 mice significantly elevated since the 3-month-old. The results indicate that LTP was an age-related reduction in APP/PS1 mice. The results of a Spearman correlation analysis showed that TMAO levels were correlated with LTP (Supplementary Figure 2).

### Age-related pathological deterioration in the hippocampus of WT and APP/PS1 mice

**Figure 3:**
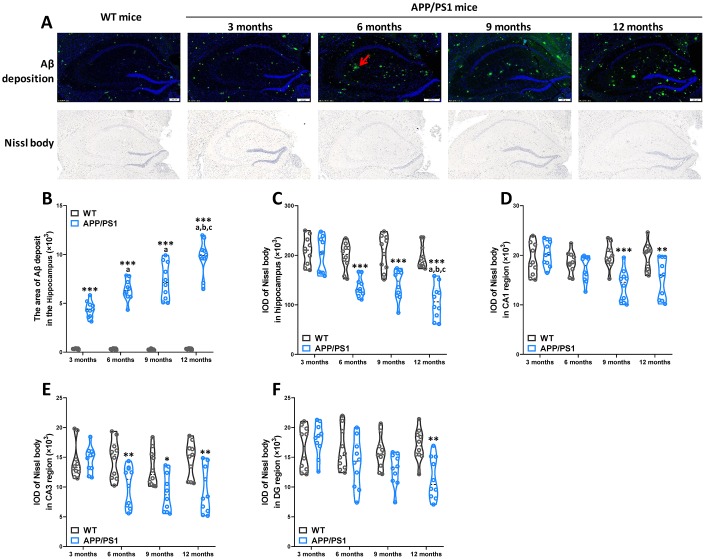
**Age-related pathological deterioration in the hippocampus of APP/PS1 mice.** Representative immunofluorescence staining images showing Αβ plaques (green and indicated by red arrows) and Nissl staining images showing Nissl bodies in the hippocampus of WT and APP/PS1 mice (**A**). Quantification of Αβ plaques(**B**) in the hippocampus of WT and APP/PS1 mice. Quantification of and Nissl bodies in the hippocampus (**C**), CA1 (**D**), CA3 (**E**) and DG (**F**) regions of WT and APP/PS1 mice. Quantitative analysis was used by Image Pro Plus 6.0 software. *P<0.05, **P<0.01, ***P<0.001, versus the age-matched WT mice, ^a^*P*<0.05, versus 3-month-old syngeneic mice, ^b^*P*<0.05, versus 6-month-old syngeneic mice, ^c^*P*<0.05, versus 9-month-old syngeneic mice by two-way repeated-measures analyses of variance with Tukey multiple comparisons tests. All values are means ± S.D. n=10.

Αβ deposition and neuronal loss in the cerebrum are pathological characteristics of AD in patients and rodent models. Our results demonstrated that APP/PS1 mice developed a critical number of Αβ plaques in the hippocampus since the 3-month-old, while Αβ plaques were not basically found in WT mice for all age groups in the present study (Figure 3A and 3B). Nissl staining showed typical neuropathological alterations in the hippocampus of APP/PS1 mice compared to WT mice (Figure 3A). Concretely, significantly lower Nissl body numbers were observed in the hippocampus (since 6 months of age), CA1 (since 9 months of age), CA3 (since 6 months of age) and DG (on 12 months of age) regions of APP/PS1 mice than in those regions of WT mice (Figure 3C–3F). The results of a Spearman correlation analysis showed that TMAO levels were correlated with Αβ plaques in the hippocampus, Nissl body numbers were observed in the hippocampus, CA1, CA3 and DG regions (Supplementary Figure 3). These data show that neuropathological changes were age-related deterioration in APP/PS1 mice.

### Global index of AD-like behavioral and pathological profile of WT and APP/PS1 mice

**Figure 4:**
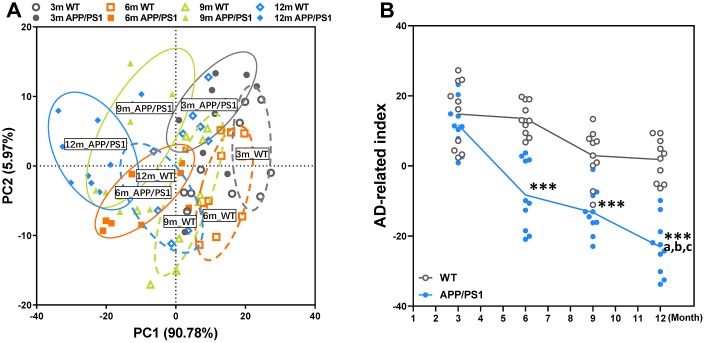
**Principal component analysis (PCA) of WT and APP/PS1 mice based on the phenotype of cognitive and pathological character.** PCA based on the data of behavioral experiment, LTP recording, immunohistochemical and Nissl staining of WT and APP/PS1 mice (**A**). Each axis was derived by principal component analysis. Each point represents one of WT and APP/PS1 mice, the number of each point represents month-age of mice. Component 1(variance explained: 90.78%), component 2 (variance explained: 5.97%) considered significant variance with a load below or equal to 0.50 (absolute value). PCA by SAS 9.2 statistics package, the significance level was set at *P* < 0.05. The mean PCA scores of WT and APP/PS1 mice (**B**). ***P<0.001, versus the age-matched WT mice, ^a^*P*<0.05, versus 3-month-old syngeneic mice, ^b^*P*<0.05, versus 6-month-old syngeneic mice, ^c^*P*<0.05, versus 9-month-old syngeneic mice by two-way repeated-measures analyses of variance with Tukey multiple comparisons tests. All values are means ± S.D. n=10.

To determine the influences of the age factor on AD-like cognition, LTP and pathological sign in APP/PS1 mice, principal component analysis (PCA) was performed here. PCA utilized data about the data of behavioral experiment, LTP recording, immunohistochemical and Nissl staining of WT and APP/PS1 mice and revealed that principal components 1 (PC1) and 2 (PC2) grouped mice from the different groups into three distinct clusters (Figure 4A). The overall behavioral and pathological profile of APP/PS1 mice that was standardized for the performance of the WT mice is presented in Figure 4B. Averaging the PCA scores obtained in each test of the battery resulted in the global index of AD-like behavioral and pathological profile of APP/PS1 mice characterized in our study. The results of a Spearman correlation analysis showed that TMAO levels were correlated with the global index of AD-like behavioral and pathological profile (Supplementary Figure 4). The index clearly shows that the AD-like characteristics of APP/PS1 mice significantly differed from that of their age-matched nontransgenic littermates since 6 months of age, and lower than other month-old APP/PS1 group.

### Age-related changes of hemostasis and circulating TMAO levels in WT and APP/PS1 mice

**Figure 5:**
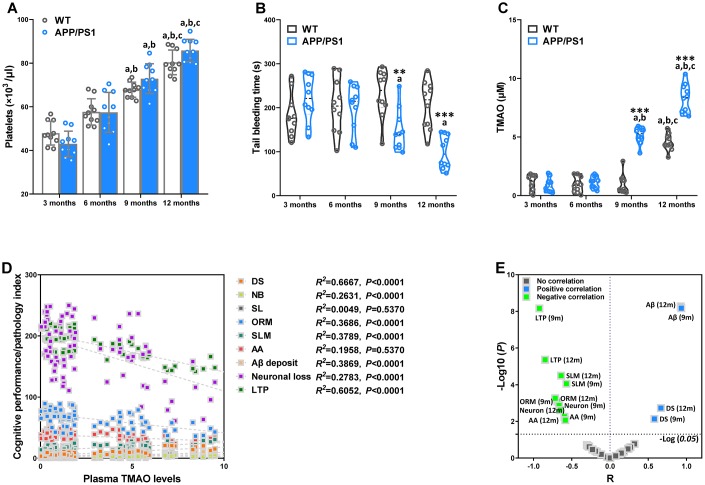
**Age-related changes of hemostasis and circulating trimethylamine-N-oxide (TMAO) levels in WT and APP/PS1 mice.** Mean count of WT and APP/PS1 mice platelets (**A**). Bleeding time measured after amputating the tail tip of WT and APP/PS1 mice (**B**). Circulating TMAO concentration in the plasma of WT and APP/PS1 mice (**C**). **P<0.01, ***P<0.001, versus the age-matched WT mice, ^a^*P*<0.05, versus 3-month-old syngeneic mice, ^b^*P*<0.05, versus 6-month-old syngeneic mice, ^c^*P*<0.05, versus 9-month-old syngeneic mice by two-way repeated-measures analyses of variance with Tukey multiple comparisons tests. All values are means ± S.D. Correlation with the TMAO levels in plasma and cognitive performance/pathology index of WT and APP/PS1 mice, as calculated via Spearman’s rank correlation coefficient (**D**). Correlation between TMAO levels and cognitive performance/pathology index of WT and APP/PS1 mice (**E**). X and Y axis was derived by R and -Log (*P*), respectively. DS means the degree of senescence, ORM means object recognition memory, SLM means spatial learning and memory, AA means active avoidance, LTP means long-term potentiation. n=10.

To study platelet function with regard to hemostasis in WT and APP/PS1 mice, blood platelet count and tail bleeding time in APP/PS1 mice of varied age were analyzed and where indicated compared to WT mice at the same age. Blood platelet count in of APP/PS1 mice did not differ significantly from the age-matched control littermates (Figure 5A). Nevertheless, the platelet count within the group significantly increased at 9 and 12 months of age in the WT and APP/PS1 group, demonstrating that platelet counts increased with age in both genotypes. The tail bleeding times of APP/PS1 and WT mice were measured after amputating the tail tip of mice (Figure 5B). Time to hemostasis at sites of a defined tail wound was served as an indicator of physiological blood clotting [19]. As shown in Figure 5B, bleeding times were significantly reduced in 9- and 12-month-old APP/PS1 mice compared with WT mice at the same age and 3-month-old APP/PS1 mice. To investigate the association between circulating TMAO and age, we analyzed plasma TMAO levels in mice of different ages (Figure 5C). As shown in Figure 5C, we found an age-related increase in circulating TMAO levels in both WT and APP/PS1 mice. In WT mice, the level of TMAO was significantly higher only at 12 months of age, while APP/PS1 mice displayed a higher level of TMAO at 9 months old that was further increased at 12 months old. The difference in plasma TMAO concentrations between WT and APP/PS1 mice was greater at 12 months old. The results indicate that circulating TMAO levels increase with age in both genotypes.

To further assess the correlations between altered plasma TMAO and AD-like cognition/LTP/pathological sign in all mice of different ages, we employed Spearman correlation analyses (Figure 5D and 5E). There was a positive correlation between elevations in plasma TMAO levels and corresponding increases in senescent degree and Αβ plaques in the 9- and 12-month-old mice. Moreover, plasma TMAO levels were inversely correlated with LTP, neuronal loss, object recognition memory, spatial learning and memory, and active avoidance in the 9- and 12-month-old mice. These results indicate increased plasma TMAO levels are associated with AD-like behavioral and pathological profile of APP/PS1 mice.

### Effects of DMB on circulating TMAO levels and cognitive impairments of WT and APP/PS1 mice

**Figure 6:**
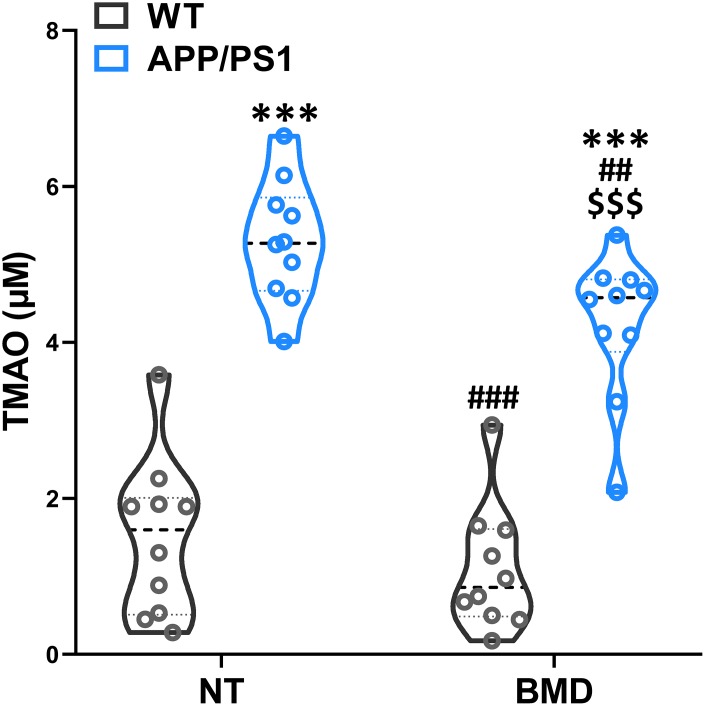
**Effects of 3,3-Dimethyl-1-butanol (DMB) on circulating TMAO levels in the plasma of WT and APP/PS1 mice.** ***P<0.001, versus the WT+ NT mice, ^##^*P*<0.01, ^###^*P*<0.001, versus the APP/PS1+ NT mice, ^$$$^*P*<0.001, versus the APP/PS1+DMB mice by one-way ANOVA analysis followed by Dunnett’s post hoc test. All values are means ± S.D. n=10. NT means no treatment.

**Figure 7:**
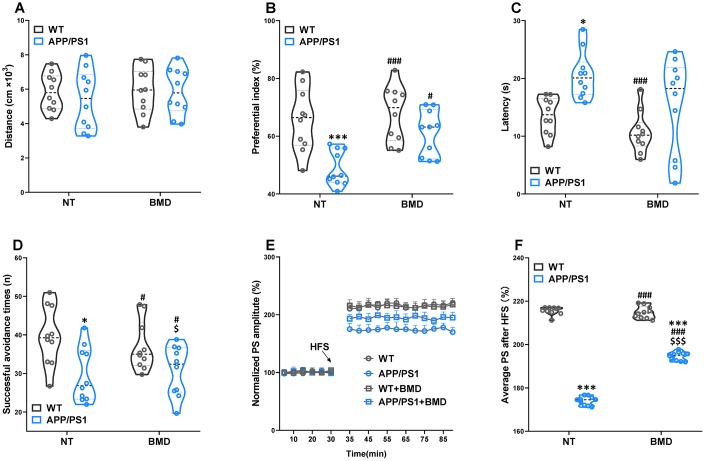
**Effects of DMB on cognitive impairments in WT and APP/PS1 mice.** The spontaneous locomotor activity (**A**), preferential index after training 1 hour in the phase of novel object test (**B**), latency in the testing task of Morris water maze test (**E**), successful avoidance times in testing phase of shuttle box test (**D**), magnitude of LTP (**E**) and summary of average PS amplitude (**F**) in WT and APP/PS1 mice. *P<0.05, ***P<0.001, versus the WT+ NT mice, ^#^*P*<0.05, ^###^*P*<0.001, versus the APP/PS1+ NT mice, ^$^*P*<0.05, ^$$$^*P*<0.001, versus the WT+DMB mice by one-way ANOVA analysis followed by Dunnett’s post hoc test. All values are means ± S.D. n=10. NT means no treatment.

After 8 weeks of DMB treatment, plasma TMAO levels were markedly higher in 9 month-old APP/PS1 mice than WT mice (Figure 6). Concomitant treatment with DMB significantly decreased plasma TMAO levels only in APP/PS1 mice compared with untreated APP/PS1 mice. In cognition aspect, the distance in spontaneous locomotor activity showed no difference among groups (Figure 7A). Moreover, the preferential index in the novel object recognition test and the successful avoidance times in the shuttle-box test of APP/PS1 mice were rescued after administration of DMB (Figure 7B and 7D). Furthermore, there was a tendency towards decreased latency in the Morris water maze test of APP/PS1 mice treated by DMB (Figure 7C). In the electrophysiology aspect, the average PS amplitude after HFS in the DMB-treated APP/PS1 mice was significantly lower than the untreated APP/PS1 mice (Figure 7E and 7F). The results of a Spearman correlation analysis showed that TMAO levels were correlated with object recognition memory, spatial learning and memory, active avoidance and LTP (Supplementary Figure 5). These results demonstrate that administration of the DMB ameliorated circulating TMAO levels and cognition deficiencies in APP/PS1 transgenic mice.

### Effects of DMB on the levels of amyloid-β in WT and APP/PS1 mice

**Figure 8:**
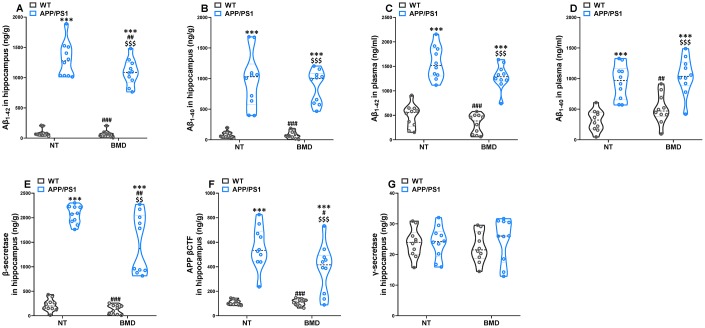
**Effects of DMB on the levels of amyloid-β (Aβ) in WT and APP/PS1 mice.** The concentration of Aβ_1-42_ and Aβ_1-40_ in the hippocampus (**A** and **B**) and plasma (**C** and **D**) of WT and APP/PS1 mice. The concentration of β-secretase (**E**), β-secretase-cleaved C-terminal fragment (βCTF) (**F**) and γ-secretase (**E**) in the hippocampus of WT and APP/PS1 mice. ***P<0.001, versus the WT+ NT mice, ^#^*P*<0.05, ^##^*P*<0.01, ^###^*P*<0.001, versus the APP/PS1+ NT mice, ^$$^*P*<0.01, ^$$$^*P*<0.001, versus the WT+DMB mice by one-way ANOVA analysis followed by Dunnett’s post hoc test. All values are means ± S.D. n=10. NT means no treatment.

The concentration of Aβ_1-42_ and Aβ_1-40_ in the hippocampus and plasma of APP/PS1 mice were assayed by AlphaLISA. The levels of Aβ_1-42_ and Aβ_1-40_ in the hippocampus and plasma of APP/PS1 mice was significantly higher than that in WT mice (Figure 8A–8D). DMB treatment led to a significant decrease in the concentration of Αβ_1-42_ in the hippocampus of APP/PS1 mice when compared with untreated APP/PS1 mice. Aβ peptides were derived from the amyloidogenic metabolism of amyloid precursor protein (APP) through its endoproteolysis by two enzymes, β- and γ-secretase. The β-secretase cut APP first to generate the N-terminus of Aβ, producing the β-secretase-cleaved C-terminal fragment (βCTF). The γ-secretase cleaved βCTF subsequently to release Aβ peptides. Different lines of evidence showed that βCTF might also contribute to the pathophysiology of AD. Hence, the concentration of βCTF, β- and γ-secretase was analyzed by ELISA (Figure 8E–8G). The concentration of β-secretase and βCTF in the hippocampus of APP/PS1 mice was significantly higher than that in WT mice. However, there was no significant difference in γ-secretase concentration of in APP/PS1 mice compared with WT mice. In APP/PS1 mice, the levels of β-secretase and βCTF in the hippocampus decreased after treatment with DMB. The results of a Spearman correlation analysis showed that TMAO levels were correlated with the levels of Aβ_1-42_ and Aβ_1-40_ in the hippocampus and plasma, β-secretase and βCTF in the hippocampus (Supplementary Figure 6). These results suggested that down-regulation of TMAO levels in plasma might selectively decline β-secretase concentration in the hippocampus of AD mice precluding the generation of Aβ peptides.

### Effects of DMB on the levels of clusterin in plasma and inflammatory status in the hippocampus of WT and APP/PS1 mice

**Figure 9:**
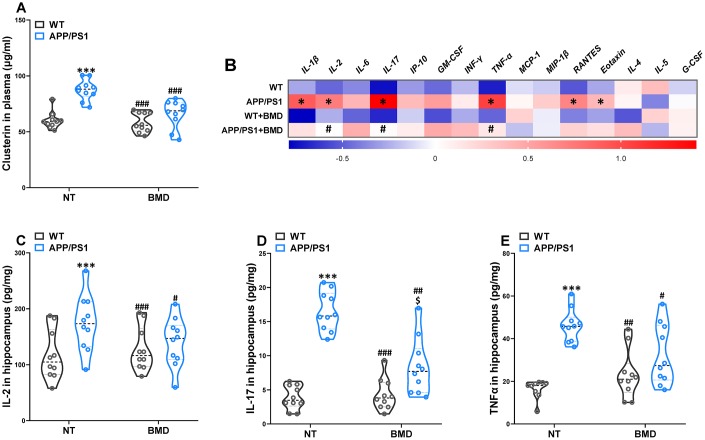
**Effects of DMB on the levels of clusterin in plasma and inflammatory status in the hippocampus of WT and APP/PS1 mice.** The concentration of clusterin in plasma (**A**). Heat map of cytokine concentrations (z-score) in the hippocampus (**B**). * represents a significant difference between WT and APP/PS1 mice, ^#^ represents a significant difference between APP/PS1 mice and APP/PS1 mice treated with DMB. The concentration of interleukin (IL)-2 (**C**), IL-17 (**D**) and tumor necrosis factor α (TNFα) (**E**) in the hippocampus. ***P<0.001, versus the WT+ NT mice, ^#^*P*<0.05, ^##^*P*<0.01, ^###^*P*<0.001, versus the APP/PS1+ NT mice, ^$^*P*<0.05, versus the WT+DMB mice by one-way ANOVA analysis followed by Dunnett’s post hoc test. All values are means ± S.D. n=10. NT means no treatment, IL means interleukin, IP means interferon-induced protein, G-CSF means granulocyte colony-stimulating factor, GM-CSF means granulocyte-macrophage colony-stimulating factor, TNF-α means tumor necrosis factor α, IFNγ means interferon-γ, MCP-1 means monocyte chemotactic protein-1, RANTES means regulated upon activation normal T cell expressed and secreted factor, MIP-1β, macrophage inflammatory protein-1β.

The abnormally elevated clusterin promoted the formation of fibrillar Aβ aggregates. The antiplatelet agent clopidogrel inhibited Aβ aggregation in transgenic AD model mice by reducing the amount of clusterin [12]. The concentration of clusterin in the plasma of APP/PS1 mice was significantly higher than WT mice (Figure 9A). In addition, DMB treatment significantly reduced the concentration of clusterin, which was significantly different from that in untreated APP/PS1 mice.

Changes in the immunological mechanisms in the brain were considered a major component of AD pathogenesis. Given the multiple links that had been ascertained between clusterin and several immunomodulatory performers, clusterin could provide some therapeutic potential as a conciliator of the abnormal immune response in the AD brain [20]. Therefore, a multiplex bead analysis was performed to detect the global inflammatory state in the hippocampus of mice (Figure 9B). We observed a significant increase in the hippocampus levels of cytokines and pro-inflammatory molecules, such as the classical mediator IL-1β, IL-2, IL-17, TNF-α, RANTES, and Eotaxin. Furthermore, reduced neuroinflammation was observed in the brains of DMB-treated mice in comparison with non-treated APP/PS1 mice, as shown by the significant reductions in IL-2 (Figure 9C), IL-17 (Figure 9D), and TNF-α (Figure 9F) levels. The results of a Spearman correlation analysis showed that TMAO levels were correlated with the levels of clusterin in the plasma, IL-2, IL-17 and TNF-α in the hippocampus (Supplementary Figure 7 and Supplementary Table 1). These results suggested that down-regulation of TMAO levels in plasma could alleviate the neuroinflammatory state of AD model mice.

## DISCUSSION

The present study reveals an important role of plasma TMAO levels in the development of cognitive and pathological deterioration in APP/PS1 mice. Results showed that plasma TMAO levels were elevated in AD transgenic mice and the elevation was observably associated with deteriorative cognitive function and AD pathology. The reductions of plasma TMAO levels induced by DMB ameliorated cognitive declines, LTP, and pathological deterioration. Meanwhile, the increases in β-secretase, βCTF, clusterin, and proinflammatory cytokines can be reversed by DMB treatment. Collectively, we hypothesized that the increased circulating TMAO levels might lead to upregulation of circulating clusterin levels, which will induce neuroinflammation and production of β-secretase and βCTF in the hippocampus, contributing to Aβ deposition and cognitive dysfunction in APP/PS1 mice.

As a meta-organismal metabolite, the generation and enrichment of TMAO are depended on bacterial and host metabolism. Intestinal microbiota metabolism of phosphatidylcholine, choline, and carnitine produces TMA, which can be used as a carbon fuel source. Then TMA will be rapidly oxidized into TMAO by host hepatic flavin monooxygenases (FMOs) in the liver [21]. In addition, plasma TMAO was not related to the strictness of both vegetarian and omnivorous diet [22, 23]. Whereas TMAO accumulation could be influenced by modifications in intestinal microbiota composition, which potentially modulates TMA generation [24]. TMAO is considered to be associated with disease pathogenesis via various mechanisms, such as enhancement of platelet hyperreactivity and thrombosis potential [25], lipid and hormonal homeostasis disruption [26], upregulation of macrophage scavenger receptors, deregulation of enterohepatic cholesterol, impairment of macrophage reverse cholesterol transport [27], and promotion of inflammation by activation of the NLRP3 inflammasome [28]. In regard to the cerebrum, TMAO has been shown to downregulate antioxidant enzyme in the hippocampus [29], induce age-related cognitive dysfunction, and accelerate the cerebral aging process [30]. In addition, TMAO is linked with the deterioration of atherosclerosis in a genetic variations modified mouse model [31], and the existence of intracephalic atherosclerosis is an independent relative risk for AD. Hence, one probable mechanism of TMAO in AD pathology is throughout the elevation of cerebral vascular disease. In the current study, we found that the plasma level of TMAO was elevated in a manner dependent on age, cognitive function and AD-like pathology in APP/PS1 mice. These results suggested that the plasma level of TMAO could be increased during the aging process. Hence, TMAO may play a role in the development of AD - related to cognitive and pathological deterioration.

Emerging evidence has revealed that the profile of gut microbial metabolites can be communicated among external contributors, nerve function and behavior [32]. Elucidating the mechanisms by which the microbial metabolites regulate nerve function and behavior are therefore of considerable importance. Elevated TMAO levels have recently been shown to be independently associated with increased pathology and neuronal degeneration in AD patients [16], which provides extra insight into intestines microbial involvement in AD. Learning and memory impairments, accompanied by disease progression of AD, are arisen from alterations in synaptic plasticity in the hippocampus. LTP, an experimental form induced by augmentation of chemical synaptic transmission, has long been suggested as a paradigm of the endogenous processes of synaptic plasticity and can influence learning and memory [18]. It has been consistently confirmed that enrichment of LTP improves cognitive performance in transgenic mice models of human neurodegeneration diseases, and vice versa [33]. In line with the previous study, we found that lower levels of plasma TMAO were detected in DMB-treated mice [34]. However, the TMAO levels in the cerebrospinal fluid of DMB-treated mice were not tested in this study. Future studies will be necessary to further investigate the effect of DMB on the TMAO levels in the cerebrospinal fluid, and whether there exists a balance between peripheral and central TMAO. By using DMB intervention, we observed that lower TMAO levels could attenuate the deterioration of novel object recognition memory, spatial memory, and active avoidance in APP/PS1 mice. Furthermore, consistently with the behavioral data, the electrophysiology experiments showed that deceased TMAO levels repaired LTP reduction in the hippocampus of APP/PS1. These results indicated that reversing the reduction of LTP might provide a neurobiological substrate for ameliorating the cognition impairments in the mice.

Aβ is a small peptide deriving from the consecutive processing of the transmembrane APP by β- and γ-secretase. As the γ-secretase cleaves at unstable sites, Aβ of various length could be generated from APP metabolism, while the most common forms in the mammalian brain are Aβ_1-40_ and Aβ_1-42_ [35]. The Aβ peptides possibly follow the hormesis rule as other endogenous molecules, for example, they are advantageous at low levels while disadvantageous at high ones [36]. The picomolar amounts of Aβ_1-42_ (physiological levels in the healthy brain) could improve hippocampal LTP, while elevated nanomolar concentrations inhibit LTP [37]. LTP and cognition can be affected by Aβ through numerous possible targets, including N-methyl-d-aspartate receptors, nicotinic acetylcholine receptors, insulin receptor, and cellular prion protein [38–41]. In our study, the concentrations of Αβ_1-42_, β-secretase, and βCTF in the hippocampus were revealed to be decreased in the DMB-treated APP/PS1 mice. Thus, our hyperthesis is TMAO could deteriorate LTP and cognition due to a significant augmentation in Αβ_1-42_ driven by increased β-secretase.

Clusterin is a multifunctional glycoprotein that contributes to the pathology, severity, and progression of AD, and could affect the toxicity and structure of Αβ [42]. Numerous clinical trials showed that multiplied plasma clusterin concentrations were associated with the clinical progression in AD [43, 44]. Clusterin has an incompatible role in AD for its anti- and pro-AD properties. Clusterin diminished Aβ accumulation and toxicity, however, when Aβ levels were higher than that of clusterin, there was an increase in Aβ amyloid formation and cytotoxicity [45, 46]. Clusterin could also regulate Aβ clearance through the intracellular uptake [47] and transportation across the blood-brain barrier [48]. In addition, fibrillar Aβ deposits in clusterin-knockout PDAPP mice (a transgenic mouse model of AD) were significantly fewer than PDAPP mice expressing clusterin. Plaques deposits in the absence of clusterin exhibited markedly reduced surrounding neuritic dystrophy, which contended in support of a pro-amyloidogenic role of clusterin in an AD mouse model [49]. More recent research in APP/PS1 mice indicated that clusterin-knockout displayed a marked decrease in Aβ deposits in the brain parenchyma, and significantly few hemorrhages and inflammation [50]. In the immunological mechanisms, the Genome-Wide Association Studies identified complement components were particular risk factors for AD [51, 52]. Within its immunomodulated functions, clusterin is well-known by regulating the complement system and multiple cytokines, including membrane attack complex [53], TGF-β [54], IL-2 [55], IL-17 [56], and TNF-α [57]. Given the multiple links that have been confirmed between clusterin and several immunomodulatory mediators of the heightened immune response in the AD brain. Therefore, the clusterin could serve as a crucial actor in our research to explore the effects of TMAO on Aβ deposition and neuroinflammation of APP/PS1 mice. In line with previously reported results, we found that high concentrations of clusterin and proinflammation cytokines were observed in APP/PS1 mice. Additionally, reduced proinflammation cytokines were observed in the hippocampus of APP/PS1 mice treated with DMB comparison with the corresponding levels in non-treated APP/PS1 mice, as shown by the significant decreases in IL-2, IL-17, and TNF-α levels.

In summary, our results indicated that the level of TMAO was associated with deteriorative cognitive function and pathology in APP/PS1 mice. DMB induced reduction of TMAO down-regulated the circulating clusterin levels, restored cytokine secretion, decreased β-secretase, βCTF, and Aβ_1-42_ levels, and ameliorated LTP, therefore improved cognition in APP/PS1 mice. However, the molecular mechanism after treatments is a limitation of our study, even though we have demonstrated that DMB treatment reduced clusterin levels in the plasma of APP/PS1 mice. Further studies are warranted to test the efficacy of DMB treatment employing various models of AD. Our findings confirmed that peripheral DMB intervention offers opportunities to regulate the cerebral pathology deterioration, and provide new insight into the effect of TMAO on AD-related cognitive dysfunction. Nevertheless, the exact molecular mechanism of peripheral TMAO mediating the Aβ deposition and cognitive dysfunction needs to be elucidated in future studies.

## MATERIALS AND METHODS

### Experimental animals

Tg (APPswe, PSEN1dE9) 85Dbo are double transgenic mice expressing a chimeric mouse/human amyloid precursor protein (Mo/HuAPP695swe) and a mutant human presenilin 1 (PS1-dE9), also known as APP/PS1 mice. Adult male APP/PS1 mice and wild type (WT) nontransgenic littermates were obtained from Huafukang bioscience company (Beijing, China) via Jackson Laboratory (Bar Harbor, ME, USA). Mice were maintained at the animal experimental center of Harbin Medical University under standard housing conditions, and fed separately. The animal feeding, administration, and experimental protocols of this study received approval from the Institutional Animal Care and Use Committee of Harbin Medical University.

For WT and APP/PS1 mice, mice were grouped by age (*n*=10), 3-month-old group, 6-month-old group, 9-month-old group, and 12-month-old group. The animals were individually subjected to behavioral tests (nest building test, locomotor activity test, novel object recognition test, Morris water maze test, and shuttle-box test), long-term potentiation (LTP) recording and bleeding time test. Following the behavioral and electrophysiological experiment, all mice were placed in a sealed chamber and euthanized via isoflurane inhalation and cervical dislocation. The hemisphere and plasma of each mouse were collected for immunofluorescence, Nissl staining, platelet counting, and TMAO analysis.

Male seven-month-old WT and APP/PS1 mice were also purchased from Huafukang bioscience company and maintained as described above. 3,3-Dimethyl-1-butanol (DMB) was an inhibitor of trimethylamine (TMA) and trimethylamine-N-oxide (TMAO) formation [58]. Seven-month-old WT and APP/PS1 mice were treated without or with 1.0% DMB (183105, Sigma-Aldrich, USA) in drinking water for 8 weeks, resulting in four experimental groups: WT (no administration, n=10), WT + DMB (n=10). APP/PS1 (no administration, n=10), APP/PS1 + DMB (n=10). The dose of DMB was chosen based on the dose in the previous study, which significantly reduced plasma TMAO in mice [58, 59]. After drug administration for 56 consecutive days, behavioral and electrophysiological tests were performed, and subsequently sacrificed for the collection of hippocampus and plasma. All samples were stored at −80 °C until use.

### Evaluation of senescence

For evaluation of the degree of senescence in the WT and APP/PS1 mice, a grading score system developed by Hosokawa, M., et al. (1984) [60] was employed. In brief, this grading system, which was designed to assess changes in animal behavior and appearance. Grade 0 was no significant changes and grade 4 was severe changes.

### Behavioral tests

### Spontaneous locomotor activity test

The spontaneous locomotor activity of each mouse was picked up for 20 minutes. Motor behavior was performed by a video-based behavior monitoring system (XinRuan Technology, China).

### Nest building test

The process for the nest building test as described previously [61]. Briefly, animals were placed into individual testing birdcages with one nestled (5-cm squares). The score of nesting was assessed at the next morning by a pre-determined measuring scale (least being 1, best being 6).

### Novel object recognition test

The object recognition test was processed as described previously [62]. The testing paradigm consists of 3 phases: habituation, training, and testing. The duration of the testing phases as detailed below. In general, To familiarize them to the testing environment, animals liberally explored a vacant chamber (10 minutes in daily) for 2 consecutive days. On the third day, mice explore two same objects (sample objects A and B) that are evenly placed at opposing ends of the chamber. Object exploration was operationally-defined as the time mice spent physically contacting the object within 0.3 cm. Each animal was allowed to explore the objects for 10 minutes. After a 1-hour training-to-testing interval, one of the familiar objects was replaced with a novel object (object C). The preferential index (time on object C/(time on object C+time on object A)×100%) was calculated to assess object recognition memory in 5 minutes testing phase.

### Morris-water maze test

Morris-water maze test proceeded in a round white pool 90 cm in diameter and 45 cm deep. The pool was filled with 30 cm of depth water. The pool temperature was maintained at 20 ± 1°C by addition of ice water. The platform was 6 cm in diameter and positioned 1 cm beneath the water surface. The training and testing sessions were 60 s in duration. In the training session, mice completed four trials daily in the presence of a hidden platform for five consecutive days. Mice that could not find the platform within 60 s were guided towards the platform and placed onto the platform for 5 s. In the testing session, the platform was removed from the pool and the mouse was allowed to search for the platform for 60 s. The latency to find the hidden platform in training and testing sessions were videoed and analyzed.

### Shuttle-box test

Shuttle box test for active avoidance was used. All avoidance training sessions consisted of 30 trials with the following parameters: 10 s tone (60 dB) and light (8W), 10 s foot shock (0.3 mA), and 15 s pause. Before the first trial of the training session, the mice were received 4 min to familiarize the testing environment. A testing session with the same parameters without foot shock, after the 5 consecutive days of training, was performed. The parameter automatically recorded was the number of successful active avoidance times.

### Bleeding time

All mice were anesthetized with ketamine (100mg/kg) and xylazine (10mg/kg). The tail was cut 3mm from the tip with a scalpel. Tail bleeding was examined by absorbing blood with filter paper once per 20 s, without making adhere to the wound site. When no bloodstain was observed on the filter paper, bleeding was deemed to have ceased. Experiments were terminated after 20 minutes.

### Platelet counting

Acid citrated dextrose-anticoagulated whole blood was diluted 1:20 in red blood cell lysis buffer (C1311, Applygen, China) for 1 minute. Platelet counts were measured by an ABX Pentra 60 Hematology Analyzer (Block Scientific, USA).

### Electrophysiology

The mice were anesthetized by intraperitoneal injection of 1.2 g/kg urethane and placed in a stereotaxic frame (NARISHIGE). A bipolar stimulating electrode was inserted in the perforant path (0.6 mm anterior to lambda, 2.4 mm to midline, 1.6-2.1 mm to brain surface). The evoked potentials were received with an electrode at the dentate gyrus (-2.0 mm from bregma, 1.0 mm to midline, 1.7-2.2 mm to brain surface). The electrical stimulus generated by a stimulator (Nihon Kohden). The pulses (1/60 Hz, 0.1 ms) transported through an isolator (Nihon Kohden) to afford a steady current. The evoked responses were augmented and low-pass filtered (1000 Hz, Axon), then transported through a data acquisition system (DIGIDATA, Axon). After obtaining a constant stimulus-response curve, a 1/3-1/2 maximum population spike (PS) was utilized. Following a 30-minute recording, the long-term potentiation was induced by high-frequency stimulation (HFS), and the PSs were recorded for 60 minutes (31-90 minutes).

### Biochemical and histochemical analyses

### Immunofluorescence

Hemispheres were stripped and immersed in 4% paraformaldehyde, then paraffin-embedded. The 5-μm-thick tissue slices were prepared. Following this, slices were incubated with mouse anti-β-amyloid antibody (6E10, 1:500, Biolegend, USA) overnight at 4 °C. The slices were incubated with fluorescein-conjugated goat anti-mouse IgG (1:50, ZSGB-Bio, China) for 4 h at room temperature. Tissue slices were photographed with a fluorescence imaging microscopy (Vectra 2, PerkinElmer-Caliper LS, USA). The area of Aβ plaque was quantified by Image Pro Plus 6.0 software.

### Nissl staining

The slices were stained using 0.5% cresyl violet acetate (Beyotime, China). The sections were scanned using a transmission electron microscope (Hitachi, Japan). The integrated optical density of Nissl bodies in the hippocampus, CA1, CA3 and DG regions was quantified by Image Pro Plus 6.0 software.

### TMAO analysis

The TMAO level was quantified using stable isotope dilution liquid chromatography-tandem mass spectrometry (LC/MS). For TMAO measurement, a 20μl plasma sample of each mouse was added to a 1.5ml Axygen tube comprising 80μl of 10μM _d9_-TMAO in methanol. The mixture was vortexed for 60s. The precipitate was incubated at -80°C for 4 hours and then centrifuged for 10 minutes at 20,000g. To calculate the TMAO concentration in plasma, various concentration standards (20μl, 0-100μM) were processed in the same way to obtain a standard curve. The standard curves were completed when *R*^2^ (coefficient of determination) reached 0.999. Supernatants were injected to a silica column (Luna 5μ Silica 100 A, Phenomenex, CA) at a flow rate of 0.4 ml/minute by an LC-20 CE Shimadzu pump system. A SIL-20AXR autosampler attached to an API 5500Q-TRAP mass spectrometer (AB SCIEX, Framingham, USA). An intermittent gradient was engendered to resolve the analyses by mixing solution A with B (solution A: 0.1% propanoic acid in water, solution B: 0.1% acetic acid in methanol) at different ratios starting from 2% B linearly to 95% B over 5 minutes, holding for 1 minute, and then decreasing back to 2% B. Analyses were monitored by electrospray ionization in positive-ion mode with multiple reaction monitoring and characteristic production transitions of TMAO at *m/z* 76-58, _d9_-TMAO at *m/z* 85-66. The stable isotope-labeled internal standard (TRIMETHYLAMINE N-OXIDE, D9, 98%) was purchased from Cambridge Isotope Laboratories (DLM-4779-PK, Andover, USA).

### Soluble Aβ analysis

The Aβ AlphaLISA assay was carried out in this research. The concentration of Aβ_1-40_ and Aβ_1-42_ in the hippocampus and plasma were measured by the human Aβ_1-40_ (AL275C, PerkinElmer, USA) and human Aβ_1-42_ (AL276C, PerkinElmer, USA) kits according to the manufacturers’ instructions.

### Enzyme-linked immunosorbent assay

The concentration of β-secretase, γ-secretase, β-secretase C-terminal fragment (βCTF) in the hippocampus and clusterin in the plasma was measured by mouse BACE-1 ELISA kit (MM-0609M1, Keta, China), mouse γ-secretase ELISA kit (MM-04110M1, Keta, China), APP beta CTF ELISA kit (MM-0610M1, Keta, China) and mouse clusterin quantikine ELISA kit (MCLU00, R&D Systems, USA) according to the manufacturer’s instructions, respectively. The absorbance was detected at 450 nm with a reference wavelength of 570 nm via an Enspire^TM^ multilabel reader 2300 (PerkinElmer, Finland).

### Multiplex bead analysis

The hippocampus supernatant was prepared according to the manufacturer’s instructions, and then diluted 1:2 in assay buffer and analyzed by a Luminex 200 (Luminex, USA). The protein expression of interleukin-1β (IL-1β), IL-2, IL-4, IL-5, IL-6, IL-17, interferon-induced protein 10 (IP-10), granulocyte colony-stimulating factor (G-CSF), granulocyte-macrophage colony-stimulating factor (GM-CSF), tumor necrosis factor α (TNF-α), interferon-γ (IFNγ), monocyte chemotactic protein-1 (MCP-1), Eotaxin, regulated upon activation normal T cell expressed and secreted factor (RANTES), macrophage inflammatory protein-1β (MIP-1β) were detected using a multiplex map kit (Mouse Cytokine/Chemokine Magnetic Bead Panel-Immunology Multiplex Assay MCYTOMAG-70K, Millipore).

### Statistical analyses

All data were expressed as means ± S.D.. GraphPad Prism 8.0 was utilized to plot and analyze partial data. Comparison of data between multiple groups was performed using a one-way analysis of variance (ANOVA) followed by a Dunnett’s post hoc test or a two-way repeated-measures ANOVA with post-hoc Tukey multiple comparisons test. Spearman correlation coefficients (R, v3.1.2) were used to measure correlations between TMAO levels in plasma and cognitive performance/pathology index. Results were considered statistically significant when *P* <0.05.

## Supplementary Material

Supplementary Figures

Supplementary Table 1
