# Supplementary material for: Decreased levels of circulating trimethylamine N-oxide alleviate cognitive and pathological deterioration in transgenic mice: a potential therapeutic approach for Alzheimer’s disease
Source: Aging (Albany NY). 2019 Oct 14;11(19):8642–63. doi: 10.18632/aging.102352 (PMC6814608; doi:10.18632/aging.102352)
Supplement: Supplementary Figures [file aging-11-102352-s002.pdf]

SUPPLEMENTARY FIGURES

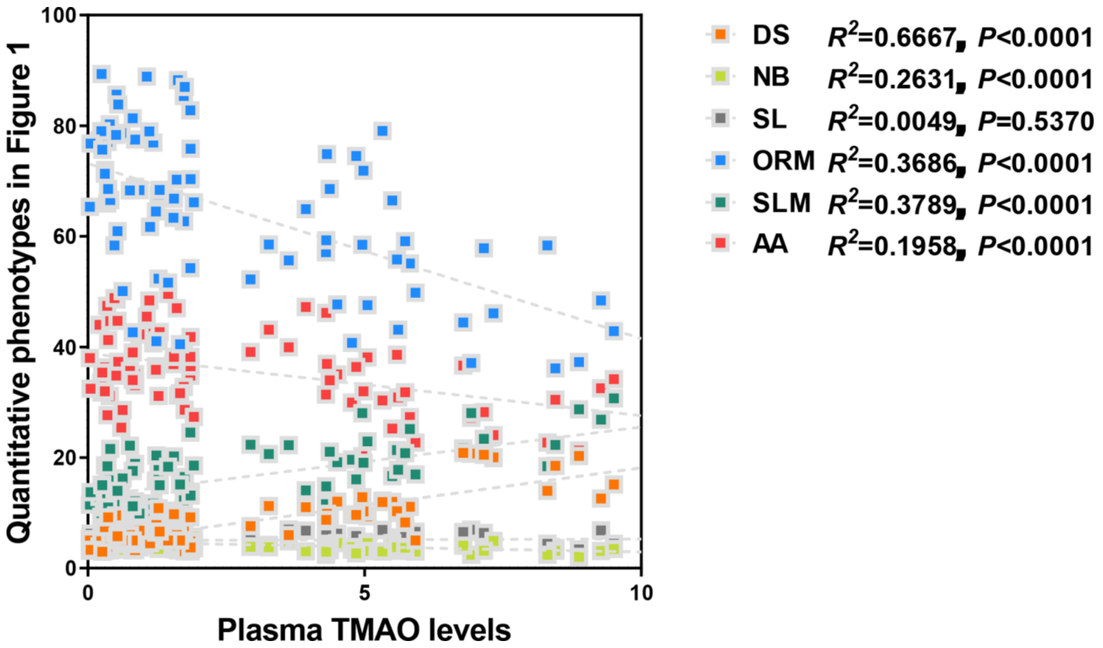

Supplementary Figure 1. Spearman correlation between the plasma TMAO concentrations and senescence / cognitive impairments in WT and APP/PS1 mice. DS means degree of senescence, NB means nest building, SL means spontaneous locomotor activity, ORM means object recognition memory, SLM means spatial learning and memory, AA means active avoidance. n=80.

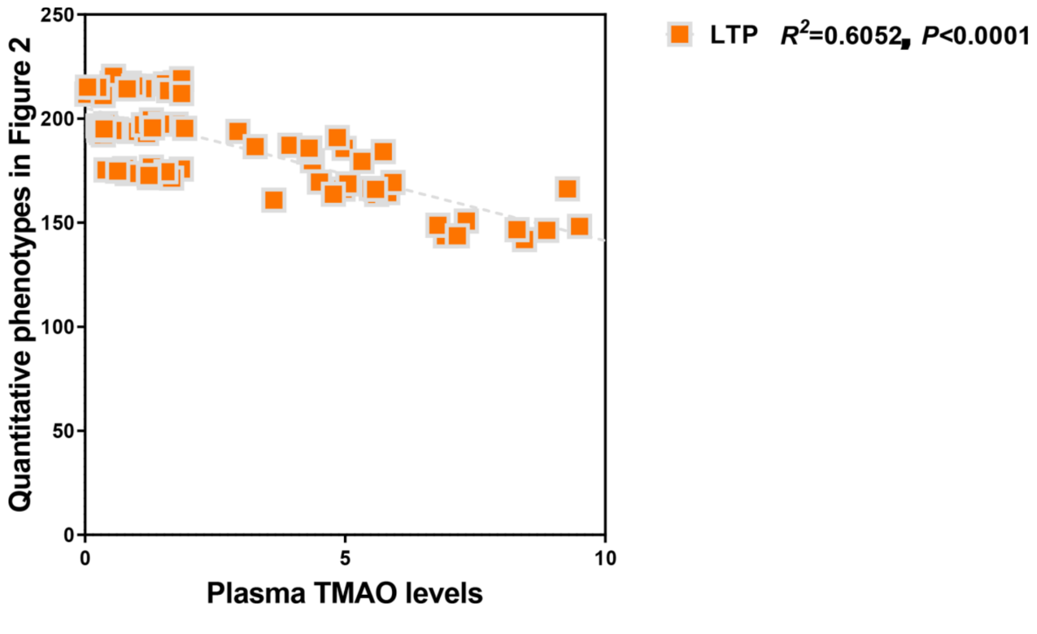

Supplementary Figure 2. Spearman correlation between the plasma TMAO concentrations and long-term potentiation in WT and APP/PS1 mice. LTP means long-term potentiation. n=80.

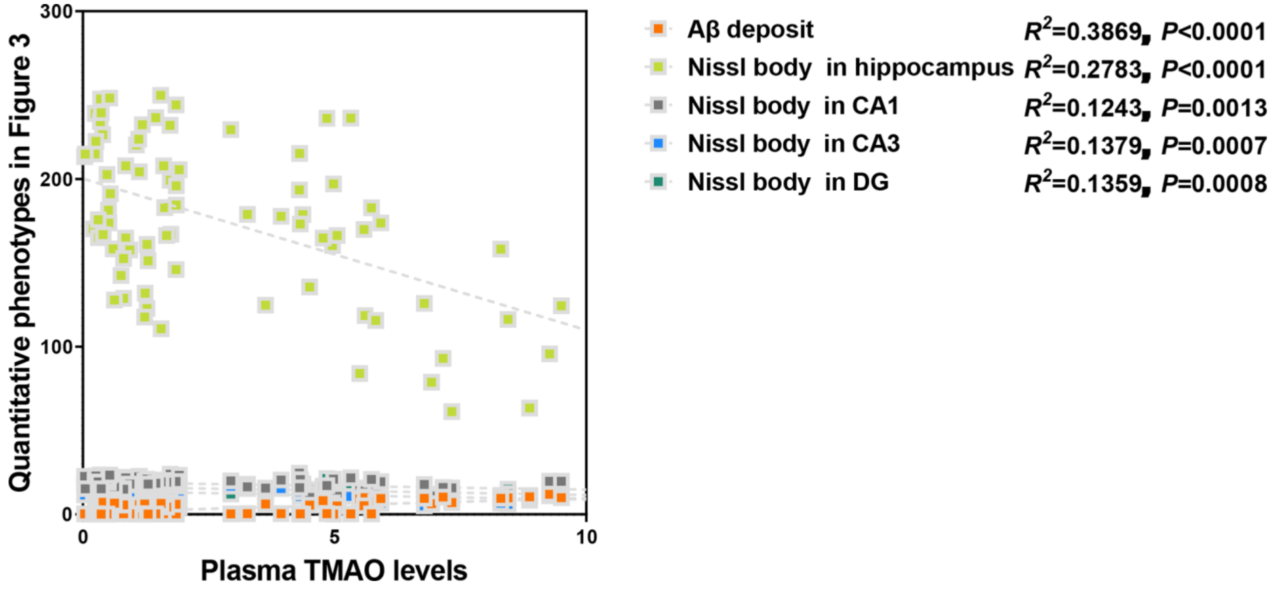

Supplementary Figure 3. Spearman correlation between the plasma TMAO concentrations and pathological deterioration in the hippocampus of WT and APP/PS1 mice. n=80.

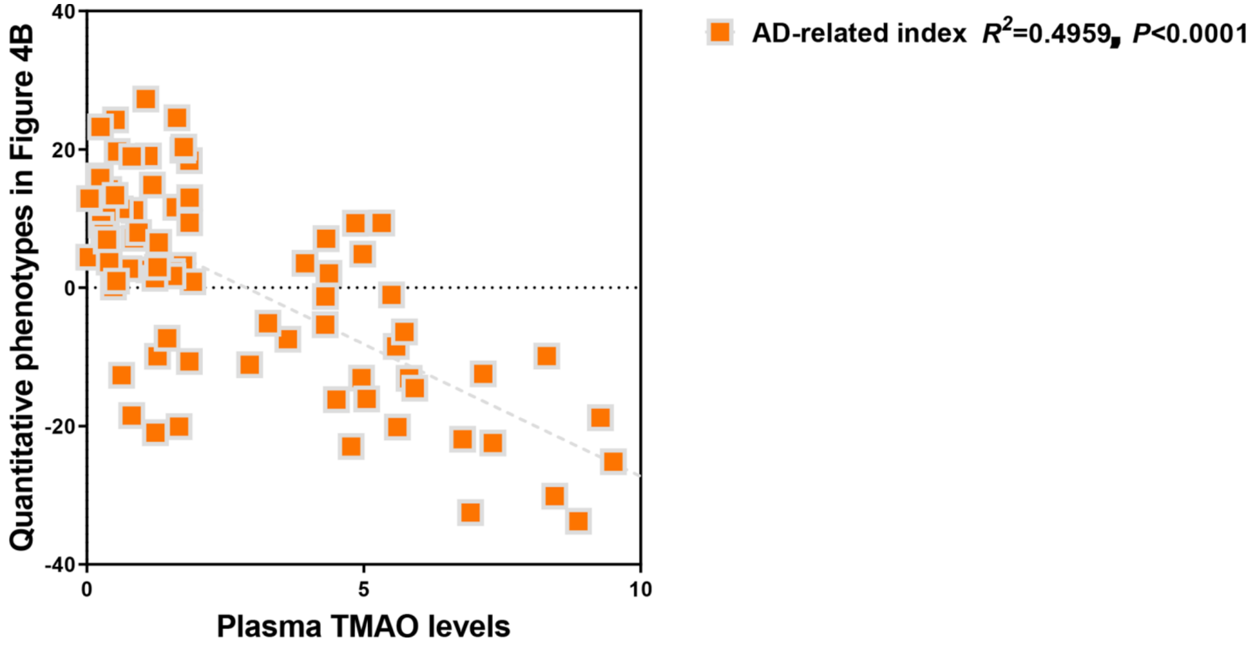

Supplementary Figure 4. Spearman correlation between the plasma TMAO concentrations and global index of AD-like behavioral and pathological profile of WT and APP/PS1 mice. n=80.

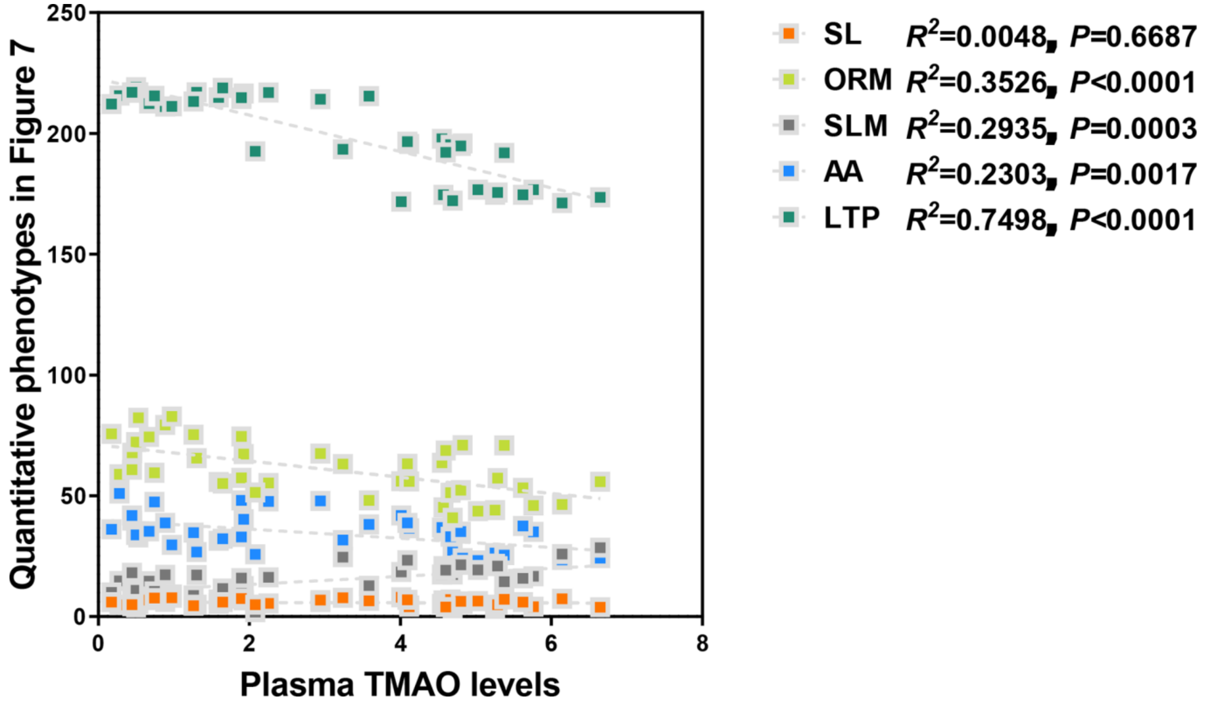

Supplementary Figure 5. Spearman correlation between the plasma TMAO concentrations and cognitive impairments in WT and APP/PS1 mice. SL means spontaneous locomotor activity, ORM means object recognition memory, SLM means spatial learning and memory, AA means active avoidance, LTP means long-term potentiation. n=40.

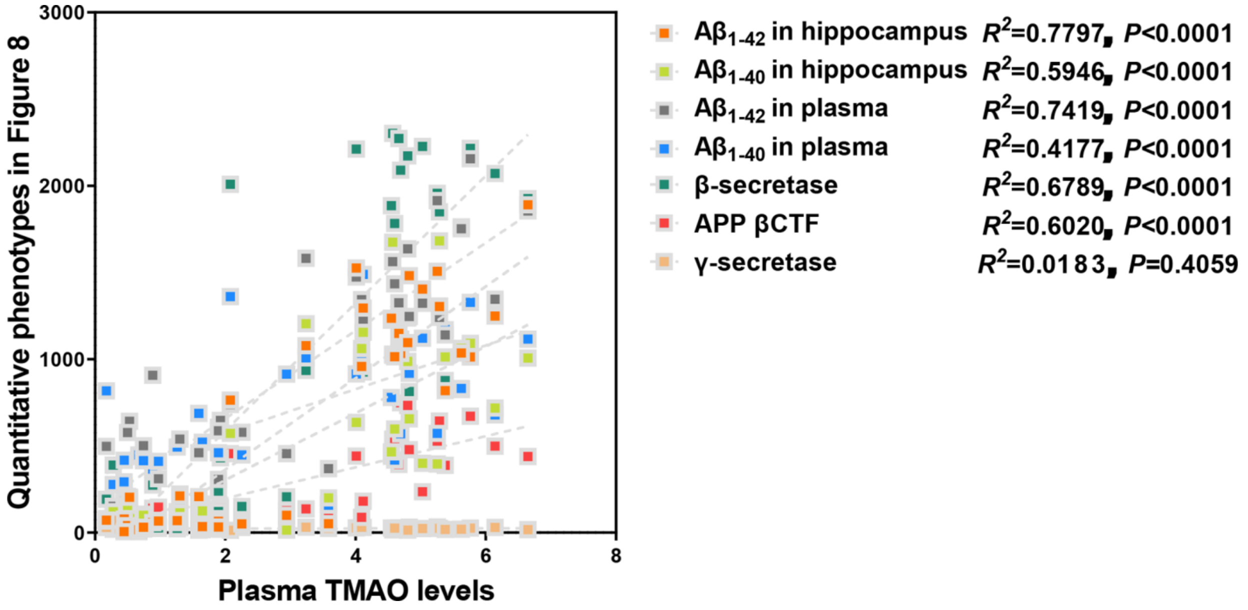

Supplementary Figure 6. Spearman correlation between the plasma TMAO concentrations and the levels of amyloid-β (Aβ) in WT and APP/PS1 mice. n=40.

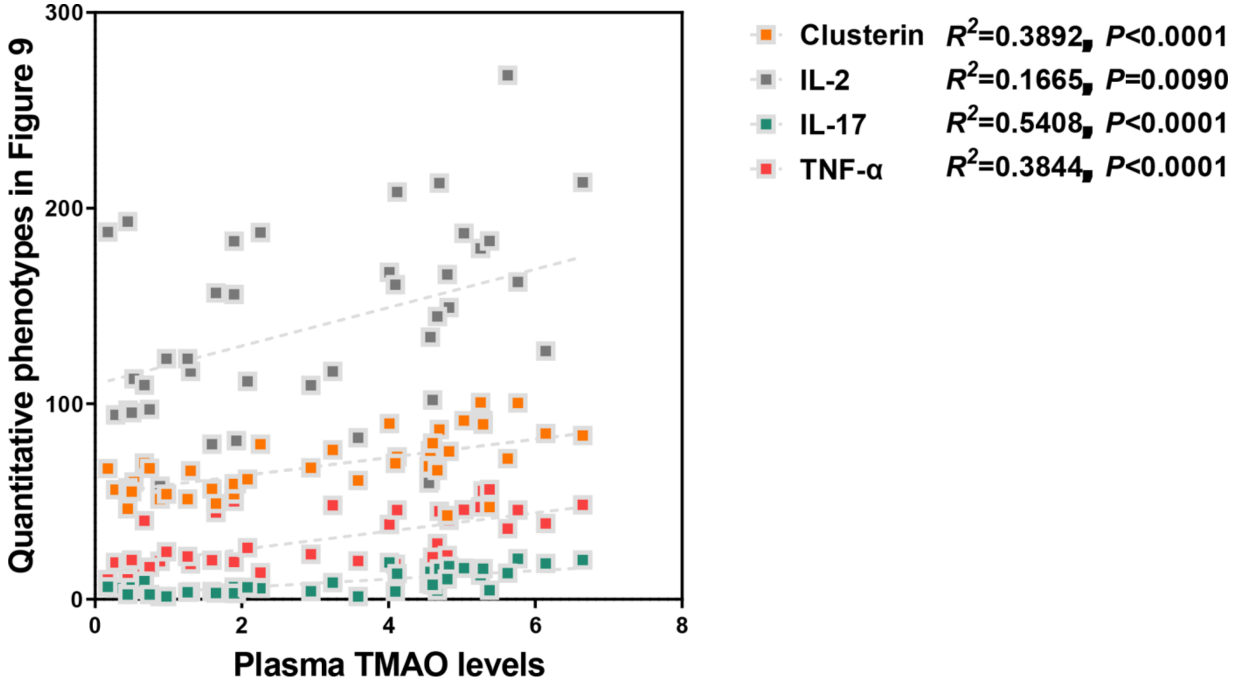

Supplementary Figure 7. Spearman correlation between the plasma TMAO concentrations and the levels of clusterin in plasma and inflammatory status in the hippocampus of WT and APP/PS1 mice. IL means interleukin, TNF-α means tumor necrosis factor α. n=40.
