# Supplementary material for: Decreased levels of circulating trimethylamine N-oxide alleviate cognitive and pathological deterioration in transgenic mice: a potential therapeutic approach for Alzheimer’s disease
Source: Aging (Albany NY). 2019 Oct 14;11(19):8642–63. doi: 10.18632/aging.102352 (PMC6814608; doi:10.18632/aging.102352)
Supplement: Supplementary Table 1 [file aging-11-102352-s001.pdf]

SUPPLEMENTARY TABLE

Supplementary Table 1. Spearman correlation between the plasma TMAO concentrations and cytokine concentrations in the hippocampus of WT and APP/PS1 mice (n=10).

	Cytokine in hippocampus											
	**IL-1β**	**IL-6**	**IP-10**	**GM-CSF**	**INF-γ**	**MCP-1**	**MIP-1β**	**RANTES**	**Eotaxin**	**IL-4**	**IL-5**	**G-CSF**
**R^2**	0.0173	0.5484	0.154	0.0938	0.3644	0.4939	0.1232	0.8682	0.1423	0.1532	0.3212	0.8893
P	0.4054	0.7900	0.6898	0.7943	0.5296	0.2699	0.8525	0.9229	0.2431	0.4802	0.6826	0.3748

IL means interleukin, IP means interferon-induced protein, G-CSF means granulocyte colony stimulating factor, GM-CSF means granulocyte-macrophage colony stimulating factor, IFNγ means interferon-γ, MCP-1 means monocyte chemotactic protein-1, RANTES means regulated upon activation normal T cell expressed and secreted factor, MIP-1β, macrophage inflammatory protein-1β.
